# Impact of the faecal immunochemical test on colorectal cancer survival

**DOI:** 10.1186/s12885-020-07074-y

**Published:** 2020-07-01

**Authors:** María Angeles Gutierrez-Stampa, Vanessa Aguilar, Cristina Sarasqueta, Joaquín Cubiella, Isabel Portillo, Luis Bujanda

**Affiliations:** 1 Osakidetza, OSI Donostialdea, Altza Primary Care; Biodonostia Health Research Institute, San Sebastián, Spain; 2 Biodonostia Health Research Institute, Red de Investigación en Servicios de Salud en Enfermedades Crónicas (REDISSEC), San Sebastián, Spain; 3 Osakidetza, Hospital Universitario Donostia, San Sebastian, Spain; 4 Gastroenterology Department, Complejo Hospitalario Universitario de Ourense, Ourense, Spain; 5 Colorectal Cancer Screening Programme, Osakidetza, Basque Health Service, Bilbao, Spain; 6 BIOEF: the Basque Foundation for Health Innovation and Research, Department of Gastroenterology, Biodonostia Institute, Avda Paseo Beguiristain s/n 20014, San Sebastián, Spain

**Keywords:** Colorectal cancer, Faecal immunochemical test, Survival

## Abstract

**Background:**

There is already evidence that the faecal immunochemical test (FIT) is a useful tool for the diagnosis of colorectal cancer (CRC) that helps to identify symptomatic patients requiring early colonoscopy. Although the recommendation to use FIT is widely accepted, there are no data concerning whether this strategy improves patient survival.The objective was to assess whether the survival is higher if CRC patients have been first diagnosed by FIT (as compared with the rest of patients with CRC).

**Methods:**

We identified all cases of CRC diagnosed between 2009 and 2016 in Donostialdea (Spain), excluding all the CRC detected in population screening. We focused on symptomatic patients. One thousand five hundred twenty-seven cases of CRC were divided into two groups based on the route to diagnosis: group 1: individuals who tested positive in a FIT during the year before diagnosis, and group 2: others.Survival was assessed by Kaplan-Meier estimation, and with the log-rank test. A Cox regression model was used to adjust for differences between groups due to other variables associated with survival.

**Results:**

One thousand nine hundred sixty-seven cases of invasive CRC were identified, of which 22.4% were detected in population screening. Of the 1527 cases diagnosed in symptomatic patients, 317 patients had undergone a FIT in the year before the diagnosis of CRC. In 279 cases(18.3%), the result had been positive and this was the first step towards their CRC diagnosis (group 1). Group 2 was composed of the 1248 cases of CRC (81.7%). Considering these cases, 1210 patients with CRC did not undergo any FIT while 38 patients presented a negative result in the year before the diagnosis. The rate of early-stage disease (stage I or II) was higher in group 1 (51.3% vs 45.5% in group 2) (*p* = 0.04). Furthermore, the 3-year survival was longer in group 1 (72% vs 59% in group 2) (HR 1.50; 95% CI 1.22–1.84).The variables independently associated with worse survival were: group 2, age > 70 years and stage at the moment of diagnosis.

**Conclusions:**

The use of FIT as a diagnostic strategy in symptomatic patients may improve survival in CRC. Nonetheless,FIT is still not widely used in our region.

## Background

Colorectal cancer (CRC) is the third most common type of cancer in Europe after breast and prostate cancer when both sexes are analysed together [1]. In Spain, in 2018, it was the type of cancer with the highest incidence in both sexes and the second cause of cancer-related death [1].

Most cases of CRC are sporadic (between 70 and 80%), but there are also heritable forms of the disease. The main risk factors, apart from family history and genetic susceptibility, are older age (over 50 years old) and being male.

On the other hand, CRC is a slow-growing cancer and is associated with hidden signs. Though there are no specific signs, the most common tend to be rectal bleeding, abdominal pain, iron deficiency anaemia, and abnormal bowel movements, as well as signs and symptoms associated with metastasis [2]. Nonetheless, symptoms are poor predictors of CRC [3, 4].

The gold standard for the detection of CRC is colonoscopy. This procedure is, however, invasive, expensive and not complication free. Therefore, it is essential to select patients with the greatest likelihood of having CRC. To help with this selection process, there is a non-invasive test, the faecal immunochemical test (FIT), which examines faecal haemoglobin concentrations (f-Hb) and has a high diagnostic accuracy for CRC [5, 6], higher than that of the SIGN or NICE criteria [7–9].

Population screening programmes have helped to diagnose CRC at early stages of the disease and decreased CRC-related mortality [10, 11]. Nonetheless, most cases of this type of cancer are still diagnosed in patients with symptoms. In recent years, it has been shown that the FIT is a test that identifies, among symptomatic patients, those with the highest risk of having CRC [12, 13] and that faecal haemoglobin is the most important factor to be considered when deciding which patients presenting in primary care with lower bowel symptoms would benefit most from referral for colonoscopy [14]. Therefore, its use has been recommended for the assessment of patients with low gastrointestinal symptoms [2, 15].

The objective of our study was to assess whether this test is used in our clinical practice and if the use of the FIT (as diagnostic tool) modifies prognosis in CRC.

## Methods

### Study population

This was a retrospective cohort study. We identified all patients, over 14 years, with CRC included in the cancer registry of Donostia University Hospital (Guipuzcoa, Spain) between 2009 and 2016. We selected patients from the health region of the Donostialdea Integrated Healthcare Organisation, which has a catchment population of 360,000 and 30 health centres.

CRC patients were classified into:

-asymptomatics patients detected in screening programme. We obtained data on population screening in the various different health centres within Donostialdea health region between 2009 and 2016. The Donostialdea population screening programme was initiated in 2009 with a biennial FIT and colonoscopy for FIT-positive individuals, targeting all 50- to 69-year-olds. In our regional screening programme, the cut-off applied is 20 μgHb/g faeces. This programme has a participation rate of 69%. By 2014, the programme had reached 100% of the population. In 2015, 85% of the population had been called for screening at least twice and 56% three times [11].

-symptomatics patients who seek medical attention for digestive symptoms: Anaemia,abnormal bowel movements,rectal bleeding, abdominal pain, anal symptoms, anorexia,...We revised all of the clinical histories of symptomatics patients with FIT performed to check the reasons for requesting the FIT .

Patients included in the study were symptomatics patients. Patients were then excluded if they had CRC in situ, cancers with histological features of a non-colon origin (melanoma, lymphoma) or CRC detected in population screening (asymptomatics). CRC was diagnosed when neoplastic cells pass through the muscularis mucosae, invading the submucosae (≥ pT1). Stage 0 Lesions, with high-grade dysplasia, intraepithelial neoplasia or intramucosal carcinoma were considered Carcinoma in situ.

We established two groups as the function of the route to CRC diagnosis. Then, we analysed a range of variables in each subgroup.

### Design and groups by route to CRC diagnosis in symptomatic patients

All symptomatic patients were allocated to one of two groups as a function of the route to diagnosis:

- Group 1: symptomatic patients with a positive FIT in the 12 months before diagnosis.

- Group 2: “others”: Symptomatic patients that either have not performed any FIT in the previous 12 months before diagnosis or displayed a negative FIT.

We identified all FIT requested between 2009 and 2016 in our health region, the laboratory at Donostia Hospital being the referral laboratory for this region. The system used for testing for occult blood in our region is the OC-Sensor® (Eiken Chemical), an immunochemical test for the specific detection of human haemoglobin with a cut-off for positivity ≥10 μg Hb/g and using a single sample. The cut-off f-Hb was as recommended in NICE DG30 [2]. Results < 10 μg Hb/g faeces were reported as f-Hb not detected. The results of this analysis are assessed qualitatively (positive or negative).

### Variables

We analysed the following variables: age, sex, histology, primary CRC site, stage at diagnosis, survival, outcome and reason for requesting the FIT. We followed up patients until 31 December 2018.

The histological variants of CRC were grouped as: adenocarcinoma, mucinous adenocarcinoma and “other” (signet ring cell carcinoma, neuroendocrine carcinoma, squamous cell carcinoma). Tumour site was defined as proximal colon (caecum, ascending colon, hepatic flexure or transverse colon), distal colon (splenic flexure, descending flexure or sigmoid colon) or rectum. The stage was defined in accordance with the TNM staging system [16]. We considered stages I and II to be early stage and stages III and IV advanced stage.

To analyse survival, patients were followed up from the date of the CRC diagnosis until death or checking their vital status on 31 December 2018. We compared 3-year survival in the two groups.

Further, a secondary analysis was performed to compare the characteristics of the CRC cases in group 2 with negative FIT results in the 2 years before the diagnosis of CRC. They were classified as false-negative FIT.

The study was approved by the Ethics Committee of Gipuzkoa (protocol code: AGS-SOH-2017-01). All the data collected in this project were processed anonymously in strict accordance with current data protection legislation (Law 41/2002 of the 14 November; Law 15/1999 of 15 December).

### Statistical analysis

A descriptive analysis of the data was performed. Qualitative variables were expressed as numbers and frequencies. The chi-squared test was used for assessing differences between qualitative variables and a binary logistic regression model was used for multivariate analysis to explore associations between group and stage. Variables with *p* values < 0.2 in the bivariate analysis were entered into the multivariate model. Survival was assessed at 3 years, by Kaplan-Meier estimation, and compared between groups with the log-rank test. A Cox regression model was used to adjust for differences between groups due to other variables associated with survival. The risk associated with each variable of interest was expressed as a hazard ratio (HR) and the corresponding 95% confidence interval (95% CI). Statistical analysis were performed with SPSS Statistics(V23) and MedCalc (v 19.2.1) .

## Results

**Fig. 1:**
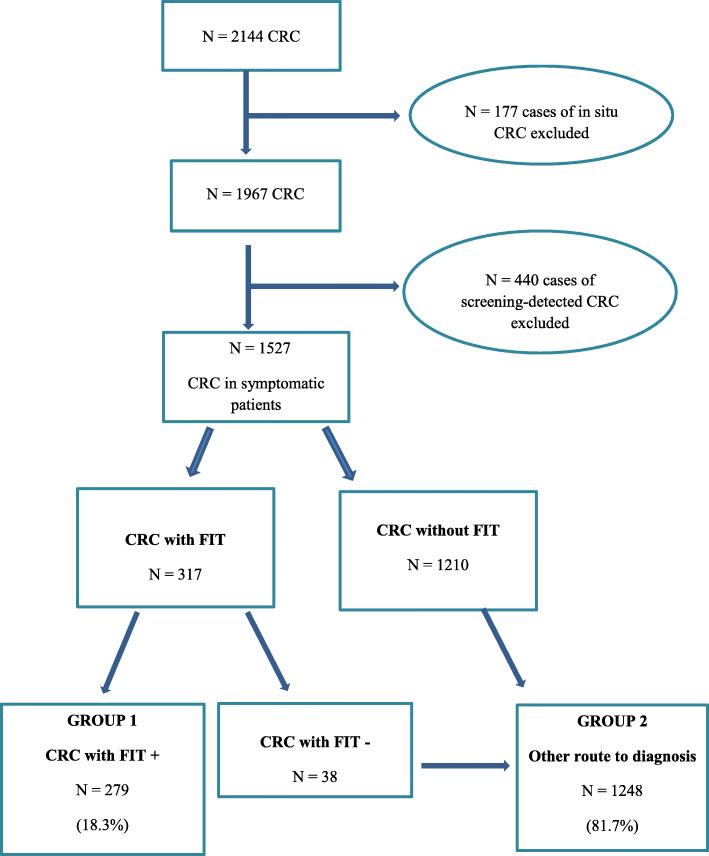
Flow of patients with colorectal cancer (CRC) though the study

Between 2009 and 2016, 2144 cases of CRC were entered in the register. The median follow-up time was 40 months (range 0–119 months). We first excluded all cases of Stage 0 disease, i.e., carcinoma in situ (*n* = 177). Subsequently, among the other cases of CRC (*n* = 1967), 440 detected in population screening (22.4%) were excluded. Finally, 1527 cases of CRC in symptomatic patients were the focus of more detailed analysis (Fig. 1).

The 440 patients with CRC detected in population screening had a mean age of 62 years and 61% of them were men. In this group, 117 tumours were located in the proximal colon, 238 in the distal colon, 81 in the rectum and 4 unknown. The CRC was detected in early stages (stages I - II) in 71.6% of cases and the 3-year survival was 93%.

### Clinical characteristics of symptomatic patients by group

Among the 1527 cases diagnosed in patients with symptoms, 317 (20.7%) patients had undergone a FIT in the year before the diagnosis of CRC. In 279 cases, the result had been positive and this was the first step towards their CRC diagnosis (group 1). Group 2 was composed of the 1248 cases of CRC (81.7%). Considering these cases, 1210 patients with CRC did not undergo any FIT while 38 patients presented a negative result in the year before the diagnosis. Patients’ clinical characteristics are summarised in Table 1.

**Table 1:** Clinical characteristics of patients by groups

	GROUP 1 *N* = 279 *N* (%)	GROUP 2 *N* = 1248 *N* (%)	p
AGE (years)			
≤ 49	13 (4.7)	52 (4.2)	
50–69	74 (26.5)	423 (33.9)	0.06
≥ 70	192 (68.8)	773 (61.9)	
SEX			
(% men)	63%	59%	0.17
SITE*			
Rectum	67 (24.1)	300 (24.0)	
Distal colon	115 (41.2)	564 (45.2)	0.31
Proximal colon	94 (33.7)	364 (29.2)	
Unknown	3(1.0)	20(1.6)	
HISTOLOGY			
Adenocarcinoma	267 (95.7)	1184 (94.9)	
Mucinous adenocarcinoma	7 (2.5)	41 (3.3)	0.8
Others**	5 (1.8)	23 (1.8)	
STAGE			
Stage I	51 (18.3)	170 (13.6)	
Stage II	92 (33.0)	398 (31.9)	
Stage III	64 (22.9)	308 (24.7)	0.04(***)
Stage IV	72 (25.8)	370 (29.7)	
Unknown	–	2(0.1)	

(Group 1: with positive faecal immunochemical test results in the previous 12 months/Group 2: patients that either did not performed any FIT in the previous 12 months before diagnosis or display a negative FIT) * Proximal colon: caecum, ascending colon, hepatic flexure or transverse colon; Distal colon: splenic flexure, descending colon and sigmoid colon. **Others: signet ring cell carcinoma, neuroendocrine carcinoma, squamous cell carcinoma. ***Chi square for linear trend

The most common reasons for requesting a FIT were anaemia (25.2%) followed by abnormal bowel movements (14.3%) (Table 2). In 33,3% of performed FIT, the reasons for requesting FIT were not registered.

**Table 2:** Symptoms for requesting FIT

	GROUP 1 ***N*** = 279 *N* (%)	NEGATIVE FIT ***N*** = 49 *N* (%)
**Anaemia**	70 (25.2)	22 (44.9)
**Abnormal bowel movements**	40 (14.3)	4 (8.2)
**Rectal bleeding**	30 (10.7)	4 (8.2)
**Abdominal pain**	23 (8.3)	4 (8.2)
**Anal symptoms, tenesmus**	17 (6.1)	2 (4.0)
**Anorexia, weight loss**	6 (2.1)	1 (2.0)
**Unknown**	93 (33,3)	12 (24.5)

(Group 1: with positive faecal immunochemical test results in the previous 12 months/ Negative FIT: patients of group 2 that display a negative FIT)

There was no significant difference between groups 1 and 2 in mean age. In both groups a larger percentage of the patients were men and the most common tumour site was the distal colon.

**Fig. 2:**
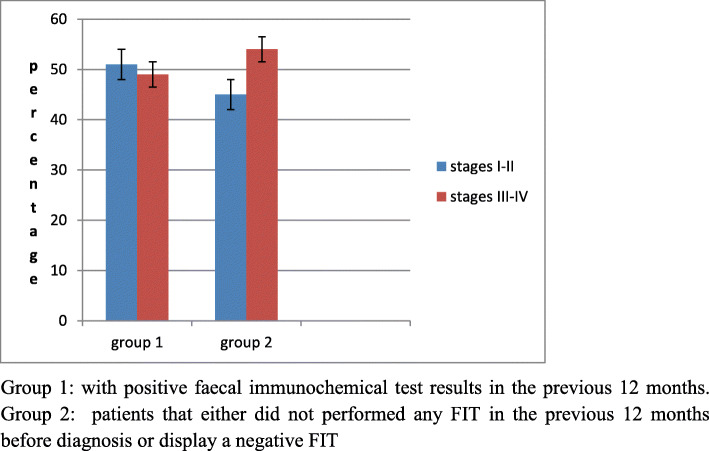
Distribution of stage of colorectal cancer by groups .Group 1: with positive faecal immunochemical test results in the previous 12 months. Group 2: patients that either did not performed any FIT in the previous 12 months before diagnosis or display a negative FIT

The distribution of cancer stage differed between the two groups (Table 1): early-stage disease accounted for 51.3% of cases of CRC in group 1 and only 45.5% in group 2 (Fig. 2).The analysis showed that patients in group 2 were 28% more likely to have advanced-stage disease, although the difference between groups was not significant (OR, 1.28; 95% CI 0.98–1.70) (Table 3).

**Table 3:** Distribution of stage of colorectal cancer by groups and other variables

	UNIVARIATE ANALYSIS			MULTIVARIATE ANALYSIS	
	EARLY STAGE (I, II) *N* = 711 N (%)	ADVANCED STAGE (III, IV) *N* = 814 *N* (%)	p	OR	95% CI
GROUP ^a^					
Group 1	143 (51.3)	136 (48.7)	0.08	1	
Group 2	568 (45.5)	678 (54.5)		1.28	0.98–1.70
AGE (years)					
≤ 49	33 (45.8)	39 (54.2)		1	
50–69	244 (49.9)	245 (50.1)	0.19	0.86	0.52–1.43
≥ 70	434 (45.0)	530 (55.0)		1.07	0.66–1.74
SITES					
Rectum	174 (47.4)	193 (52.6)			
Distal colon	310 (45.7)	368 (54.3)	0.71	–	–
Proximal colon	220 (48.1)	237 (51.9)			
Unknown	7(30.4)	16(69.6)			
HISTOLOGY					
Adenocarcinoma	683 (47.1)	766 (52.9)		1	0.64–2.04
Mucinous adenocarcinoma	21 (43.7)	27 (56.3)	0.06	1.14	1.15–6.46
Others^b^	7 (25.0)	21 (75.0)		2.72	

*OR* odds ratio, *CI* confidence interval ^a^Group 1; with positive faecal immunochemical test results in the previous 12 months. Group 2: patients that either did not performed any FIT in the previous 12 months before diagnosis or display a negative FIT ^b^Others: signet ring cell carcinoma, neuroendocrine carcinoma, squamous cell carcinoma.

### Mortality

**Fig. 3:**
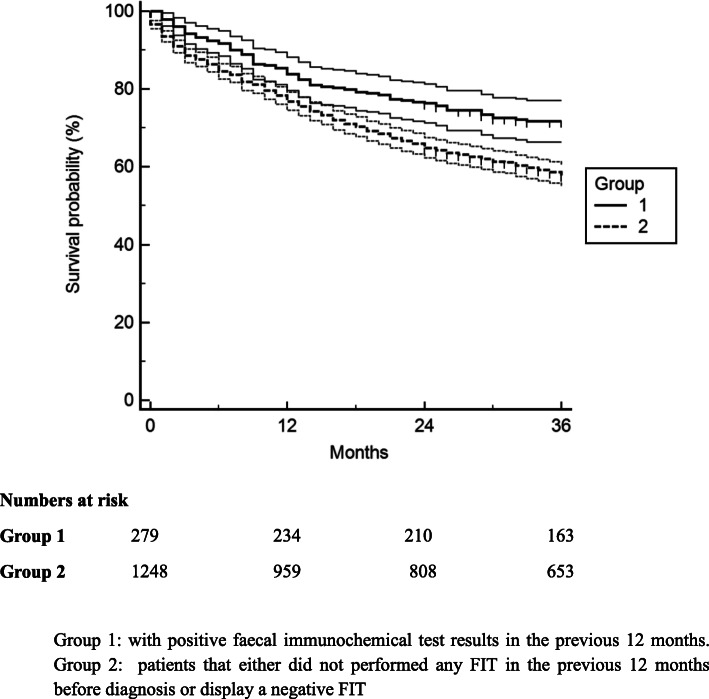
Kaplan-Meier overall survival curves by group with 95% confidence intervals and numbers at risk. Group 1: with positive faecal immunochemical test results in the previous 12 months. Group 2: patients that either did not performed any FIT in the previous 12 months before diagnosis or display a negative FIT

The 3-year survival in CRC was 72% (95% CI;66–78) in group 1 and 59%(95% CI;56–62) in group 2 (*p* < 0.0005; (Fig. 3). After adjusting for other factors associated with survival, namely, histology, age and stage, the difference in survival was smaller but remained significant (HR 1.50; 95% CI 1.22–1.84) (Table 4). The variables independently associated with survival were: group 2, age > 70 years and stage at the moment of diagnosis (Table 4).

**Table 4:** Three-year CRC survival as a function of different factors (univariate and multivariate analysis)

	UNIVARIATE ANALYSIS			MULTIVARIATE ANALYSIS	
	% Three-year CRC survival	Standard error	P	HR	95% CI
GROUPS^a^					
Group 1	72	0.03	< 0.0005	1	
Group 2	59	0.01		1.50	1.22–1.84
AGE (years)					
≤ 49	80	0.05		1	
50–69	72	0.02		1.35	0.83–2.19
≥ 70	54	0.02	< 0.0005	2.96	1.85–4.75
SITES					
Rectum	57	0.03			
Distal colon	62	0.02	< 0.25		
Proximal colon	63	0.02			
HISTOLOGY					
Adenocarcinoma	61	0.01		1	
Mucinous adenocarcinoma	73	0.06	0.18	0.53	0.34–0.83
Others^b^	42	0.09		1.32	0.81–2.14
STAGE					
Stage I	92	0.02		1	
Stage II	82	0.02	< 0.0005	1.32	0.97–1.79
Stage III	67	0.03		2.18	1.61–2.95
Stage IV	18	0.02		8.83	6.63–11.77

*HR* hazard ratio, *CI* confidence interval ^a^Group 1; with positive faecal immunochemical test results in the previous 12 months. Group 2: patients that either did not performed any FIT in the previous 12 months before diagnosis or display a negative FIT ^b^Other: signet ring cell carcinoma, neuroendocrine carcinoma, squamous cell carcinoma

We also analysed the survival between the group 1 and the group 2 but without the 49 false-negative FIT results and the 3-year survival in CRC was 72% in group 1 and 58% in group 2 (*p* < 0.0005).

### False-negative FIT results

In group 2, 38 CRC cases with negative FITs were detected in the year before diagnosis and 11 patients with a negative FIT were identified in the previous year. Overall, 49 false-negative FIT results were identified in group 2 in the 24 months before diagnosis. The most common reason for requesting a FIT in patients with false-negative results was anaemia (40.3%) (Table 2). The characteristics of the patients are summarised in Table 5. Patients with CRC who had had a negative FIT were likely to have disease in the proximal colon (OR 3.57; 95% CI 1.27–10.03) and at stage III (OR 4.1; 95% CI 1.1–15.36), and 51% of them were women. Although 57.1% of these patients had advanced-stage disease at diagnosis, differences in survival compared to that in group 1 did not reach significance.

**Table 5:** Clinical characteristics of colorectal cancer with a negative FIT(of group 2) vs Group 1

	UNIVARIATE ANALYSIS			MULTIVARIATE ANALISIS	
	GROUP 1 N = 279 N (%)	NEGATIVE FIT (of group 2) N = 49 N (%)	P	OR	95% CI
AGE (years)					
≤ 49	13 (4.7)	1 (2)			
50–69	74 (26.5)	17 (34.7)	0.4		
≥ 70	192(68.8)	31 (63.3)			
SEX					
Men	177(63.4)	24 (49)	0.05	1	
Women	102(36.6)	25 (51)		1.8	0.95–3,4
SITES					
Rectum	67 (24.0)	5 (10.2)		1	
Distal	115(41.2)	17 (34.7)	0.03	2.01	0.70–5.78
Proximal	94(33.7)	24 (49.0)		3.57	1.27–10.03
Unknown	3(1.1)	3(6.1)			
HISTOLOGY					
Adenocarcinoma	267(95.7)	46 (93.9)			
Mucinous adenocarcinoma	7 (2.5)	2 (4.1)	0.8		
Others^a^	5 (1.8)	1(2)			
STAGE					
Stage I	51 (18.3)	3 (6.1)		1	
Stage II	92(33)	18 (36.7)	0.18	2.87	0.79–10.4
Stage III	64(22.9)	15 (30.6)		4.1	1.10–15.36
Stage IV	72 (25.8)	13 (26.5)		2.82	0.74–10.69
Overall 3-year survival	72%	63%	0.5		

Group 1:colorectal cancer with a positive FIT/ Negative FIT of Group 2: patients of group 2 that display a negative FIT. *OR* Odds ratio; *CI* confidence interval;^a^Others: signet ring cell carcinoma, neuroendocrine carcinoma, squamous cell carcinoma

## Discussion

### The main findings of our study

The use of FIT in symptomatic patients is associated with a better prognosis in CRC. Three-year survival was greater in the CRC group diagnosed after a positive FIT (72% vs 59%). The rate of early-stage disease was also higher in this group (51.3%) than in the group 2 (45.5%). Nonetheless, this test is still not widely used in primary care consultations in our region (having been requested for only a fifth of all symptomatic CRC patients).

There is already evidence that FIT is a useful tool for the diagnosis of CRC that helps to identify symptomatic patients requiring early colonoscopy [4–7, 17]. In fact, the diagnostic guidance (DG30) of the National Institute for Health and Care Excellence (NICE) and other clinical practice guidelines recommend its use for the assessment of patients with lower gastrointestinal symptoms [2, 15]. Despite this, according to our results, the FIT had been used as a diagnostic tool by general practitioners only in 20.7% (*n* = 317) of all the cases of CRC diagnosed in symptomatic patients, and only 18.3% (*n* = 279) of cases of CRC were diagnosed after a positive FIT. These figures demonstrate the low rate of adoption of this recommendation in our setting. In our health system, the FIT has been rolled out progressively in parallel with the screening programme initiated in 2009; however, its rate of adoption in primary care has been uneven and generally poor.

On the other hand, although the recommendation to use FIT is widely accepted, there are no data concerning whether this strategy improves patient survival. Our study indicates a better prognosis in CRC diagnosed after a positive FIT. In these cases, the disease was diagnosed at a localised stage in 51.3% of cases (vs 45.5% in the other group) and the 3-year survival was significantly greater (72% vs 59%), despite the fact that a higher percentage of those with a positive FIT were over 70 years of age.

One of the factors that may explain the better prognosis in CRC after a positive FIT is the shorter time to diagnosis. If patients who seek medical attention with unspecific symptoms undergo a FIT, rather than just having their condition monitored, it would be possible to reduce the time to diagnosis, on the one hand, because FIT has been carried out early, and on the other, because if the test is positive the patient is referred for urgent colonoscopy. In our study, we found that 75.1% of patients with CRC detected after a positive FIT were diagnosed within 3 months (from the FIT test results to histological diagnosis). It is already known that repeat primary care consultations lengthen the time to diagnosis [18, 19] and that diagnostic delay is one of the most important factors in terms of survival [20]. In this context, a FIT may be helpful in that it speeds up decisions on the clinical management of these patients. Another explanation would be that patients at more advanced stages have more severe symptoms and that, in these cases, general practitioners refer them directly to a gastroenterologist or even a hospital emergency department, while when symptoms are milder, and given the simplicity of the FIT, the test is requested to rule out the presence of CRC with certainty. Finally, it could be that some of these positive FIT are result of opportunistic screening because in 33,3% of performed FIT the reasons for requesting FIT were not registered.

### Strengths and weaknesses of our study

The greatest strength of this research is that it is the first study that analyses the impact of the use of FIT on CRC survival in symptomatic patients, compared to other patients with CRC. To our knowledge, no previous studies have analysed whether the use of FIT in consultations, as a diagnostic test for symptomatic patients, has changed prognosis in CRC. Further, we should highlight that the study was carried out at population level. We identified all the patients with CRC from a registry of tumours at Donostia Hospital in the period 2009–2016, and we selected all the patients in the catchment area of the Donostialdea Integrated Healthcare Organisation. On the other hand, few studies have analysed, among all cases of CRC, the percentage detected by different routes and impact of route to diagnosis on prognosis in this disease.

Nonetheless, we recognise that our study has some limitations. Since it was a retrospective study, we were not able to assess patient comorbidities or other risk factors such as personal or family background. We do not know which factors related to patients or doctors could influence the decision of requesting the FIT or not. We were not able to determine accurately how group 2 patients were diagnosed (through the emergency department, inpatient wards or primary care consultations) or the time between symptom onset and diagnosis. Therefore,we have some limitations of making conclusions on causality because there may be biases in estimates due to residual confounding.

### Our study compared with other research

Our data reflect that, as observed in other studies, the incidence of CRC is higher in men and at older ages, CRC being uncommon in under-55-year-olds (3.3%). The most common site is the distal colon and the most common histological type is adenocarcinoma.

According to our results, only 22.4% of all cases of CRC are detected in population screening. Most cases of CRC are detected in symptomatic patients. A study in Scotland found that 18% of cases of CRC were detected in population screening [21]. Unlike the Scottish programme, which used a guaiac-based test, in the Basque Country, the population screening programme is based on the FIT. Here, there is a high participation rate (69%), exceeding that recommended in European guidelines (65%), and 92% of those referred agree to colonoscopy, and despite this, 53% of cases of CRC detected in the screening-eligible age range (50–69 years) were diagnosed in symptomatic patients. This implies that we need to further increase the rate of participation in population screening. On the other hand, according to our data, 15.4% (304) of all the cases of CRC were diagnosed in individuals between 70 and 75 years old, and 55.1% of these had advanced-stage disease. According with others studies which show a high incidence of CRC in patients with more than 74 years [22], in order to improve the screening program, it is pivotal to consider the screening in the elderly. Therefore, we believe that if we extended the upper age limit for the screening programme, we would be able to increase the percentage of diagnoses made at earlier stages of the disease, and in turn, improve survival. It is already known that population screening programmes for CRC are associated with a reduction in mortality due to CRC [10, 11].

On the other hand, although FIT has a very high diagnostic accuracy for detecting CRC, [3, 8] in our study, we detected 49 cases of CRC in patients who had had negative FIT in the 24 months before the diagnosis. Therefore, these results indicate, in line with meta-analyses [12, 13, 23], that FIT is not a diagnostic test by itself but it is rather a diagnostic support tool that should be used together with clinical assessment of patients.

Various studies in the literature have described the clinical characteristics of interval cancers within screening programmes [24], but to our knowledge, none have analysed the clinical characteristics of cases of CRC in symptomatic patients who have had a negative FIT result. In our study, patients with CRC who had had a negative FIT were more likely to have disease in the proximal colon and at stage III, and to be a woman. Nonetheless, we did not observe statistically significant differences in survival, despite 57.1% of the patients being diagnosed at an advanced stage. These clinical characteristics are similar to those of interval cancers from population screening programmes [25]. Such tumours have often been found in patients with genetic abnormalities linked to Lynch syndrome, which is associated with a better prognosis. Nonetheless, our results are limited by the relatively small sample size. Further studies are required to confirm these results.

### Implications for clinicians and managers

The FIT is useful as a simple, cheap diagnostic test that can be requested by primary care doctors and it is recommended by guidelines for the assessment of patients with digestive symptoms [2, 13]. It serves to select patients who should be referred for urgent colonoscopy [26] and together with a patient’s medical history and a physical examination allows significant colorectal disease to be ruled out, avoiding unnecessary colonoscopies [27, 28]. Moreover, some research has shown that if a FIT is used, along with other parameters such as age and gender (FAST score), rather than the criteria proposed by NICE, 42% more cases of CRC are detected [29]. Recent reports have shown that FIT is considered the most important parameter for the detection of CRC, among all the factors that are usually taken into account [30].

On the other hand, the better survival observed in our study in cases of CRC detected after a positive FIT suggests that it may be a useful diagnostic tool for the early detection of CRC. Given all these factors, it seems that this test should be more widely adopted in routine clinical practice, above all considering how little it is currently used by primary care doctors.

Nonetheless, there is a need for further research with larger samples sizes to confirm our results and, if they are confirmed, investigate the factors underlying the better prognosis.

## Conclusion

The use of FIT in symptomatic patients may improve prognosis in CRC. Nonetheless, this type of test is still not widely used in our clinical practice.

## Data Availability

The datasets during and/or analysed during the current study available from the corresponding author on reasonable request.

## Abbreviations

CI: Confidence interval
CRC: Colorectal cancer
FIT: Faecal immunochemical test
HR: Hazard ratio
OR: Odds ratio
f-Hb: Faecal haemoglobin concentrations
